# Increased Risk of Hemorrhagic Stroke in Patients with Migraine: A Population-Based Cohort Study

**DOI:** 10.1371/journal.pone.0055253

**Published:** 2013-01-25

**Authors:** Cheng-Ya Kuo, Ming-Fang Yen, Li-Sheng Chen, Ching-Yuan Fann, Yueh-Hsia Chiu, Hsiu-Hsi Chen, Shin-Liang Pan

**Affiliations:** 1 Department of Physical Medicine and Rehabilitation, National Taiwan University Hospital and National Taiwan University College of Medicine, Taipei, Taiwan; 2 School of Oral Hygiene, College of Oral Medicine, Taipei Medical University, Taipei, Taiwan; 3 Department of Nutrition and Health Sciences, Kainan University, Taoyuan, Taiwan; 4 Department and Graduate Institute of Health Care Management, Chang Gung University, Tao-Yuan, Taiwan; 5 Centre of Biostatistics Consultation, College of Public Health, National Taiwan University, Taipei, Taiwan; 6 Division of Biostatistics, Graduate Institute of Epidemiology, College of Public Health, National Taiwan University, Taipei, Taiwan; FuWai hospital, Chinese Academy of Medical Sciences, China

## Abstract

**Objective:**

Previous studies on the association between migraine and the risk of developing hemorrhagic stroke (HS) have generated inconsistent results. The aim of the present population-based, age- and sex- matched follow-up study was to investigate whether migraine is associated with an increased risk of HS.

**Method:**

A total of 20925 persons with at least two ambulatory visits in 2001 with the principal diagnosis of migraine were enrolled in the migraine group. The non-migraine group consisted of 104625, age- and sex- matched, randomly sampled subjects without migraine. The two-year HS-free survival rates for these 2 groups were estimated using the Kaplan-Meier method. Cox proportional hazards regression was used to estimate the effect of migraine on the occurrence of HS.

**Results:**

During the 2 year follow-up, 113 subjects in the migraine group (0.54%) and 255 in the non-migraine group (0.24%) developed HS. The crude hazard ratio (HR) for developing HS in the migraine group was 2.22 compared to the non-migraine group (95% confidence interval [CI]: 1.78–2.77, *p*<0.0001) and the adjusted HR was 2.13 (95% CI: 1.71–2.67, *p*<0.0001) after controlling for demographic characteristics and comorbid medical disorders.

**Conclusions:**

This population-based age- and sex- matched cohort study shows that migraine was linked to an increased risk of HS.

## Introduction

Migraine is a neurological disease characterized by recurrent episodes of headache [1]. The reported prevalence in men and women is 8.6% and 17.5%, respectively [2]. While migraine has been associated with an increased risk of ischemic stroke [3]–[5], it remains controversial whether it is also linked to an increased risk of hemorrhagic stroke (HS). While retrospective studies have suggested a link between migraine and HS in women [6], [7] and prospective data from the Women's Health Study showed an increased risk of HS in migraineurs with aura [8], several other studies were unable to identify a higher risk of HS in patients with migraine [5], [9], [10]. Such inconsistency may be partly due to small sample size and partly to the retrospective case-control design adopted in the majority of studies, which makes it difficult to establish a temporal relationship between migraine and HS. In addition, research on the association between migraine and HS in men is sparse. We therefore performed this large-scale, population-based, age- and sex-matched follow-up study to investigate whether migraine is associated with increased risk of developing HS.

## Materials and Methods

### Data source

The data used in this study were obtained from the complete National Health Insurance (NHI) claim database in Taiwan for the period 2000 to 2003. The NHI program has been implemented in Taiwan since 1995, and the coverage rate was 96% of the whole population in 2000 and 97% at the end of 2003, at which time more than 21.9 million inhabitants were enrolled. It should be noted that the rationale for using the NHI database after 2000 is that, from Jan 1, 2000, according to the rules of the Bureau of NHI, the NHI claim data were all encoded using the standardized International Classification of Disease, 9^th^ Revision, Clinical Modification (ICD-9-CM). To keep individual information confidential in order to satisfy regulations on personal privacy in Taiwan, all personal identification numbers in the data were encrypted by converting the personal identification numbers into scrambled numbers before data processing. Because the database used consists of de-identified secondary data released for research purposes, this principle complies with the Personal Information Protection Act in Taiwan, and this study was exempt from full review by the National Taiwan University Hospital Research Ethics Committee.

### Study subjects and design

We used a prospective age- and sex- matched cohort study to investigate the effect of migraine on the risk of developing subsequent HS. The study population consisted of a migraine group and a non-migraine group, both selected from Taiwanese residents in the complete NHI claims database for 2001, in which more than 21.6 million persons were registered.

The migraine group consisted of subjects who had received a principal diagnosis of migraine (ICD-9-CM codes 346) during ambulatory medical care visits between January 1, 2001 and December 31, 2001. The index visit was defined as the first ambulatory visit during which a principal diagnosis of migraine was made. To maximize case ascertainment, only patients who had at least 2 ambulatory visits (including the index visit) with the principal diagnosis of migraine in this period were initially considered for inclusion in the migraine group (n = 44825). The exclusion criteria for the recruitment of subjects into the migraine group were: (1) age less than 18 years (n = 2394) to restrict the research sample to the adult population; (2) a previous diagnosis of migraine (ICD-9-CM codes 346) during 2000 (n = 19550) to increase the likelihood of identifying only new incident migraine cases in 2001; and (3) a previous diagnosis of any type of stroke (ICD-9-CM codes 430 – 438) before the index visit (n = 4210). A total of 20925 subjects was therefore included in the final migraine group.

The non-migraine group was taken from the remaining subjects without a diagnosis of migraine in the same 2001 NHI claim database. The first ambulatory medical care visit during 2001 was assigned as the index visit. The exclusion criteria for recruiting subjects into the non-migraine group were: (1) a previous diagnosis of migraine (ICD-9-CM codes 346) before the index visit and (2) a previous diagnosis of any type of stroke (ICD-9-CM codes 430–438) before the index visit. We randomly sampled 5 persons for each subject in the migraine group, matched by age and sex. A total of 104625 subjects was included in the non-migraine group.

### Outcome and covariates

All ambulatory medical care records and inpatients records for each subject in the migraine and non-migraine groups were tracked from their index visit for a period of 2 years. The mortality data for the subjects who died during the follow-up were obtained from the mortality registry. The date of the first occurrence of a principal diagnosis of HS (ICD-9-CM codes 430, 431, or 432) within the follow-up period was defined as the primary endpoint. All subjects were followed from the index visit to the first occurrence of HS, death, or end of follow-up. The data for pre-existing cardiovascular comorbidities, including diabetes mellitus (ICD-9-CM code 250), hypertension (ICD-9-CM codes 401–405), hyperlipidemia (ICD-9-CM code 272), coronary heart disease (ICD-9-CM codes 410–414, and 429.2), chronic rheumatic heart disease (ICD-9-CM codes 393–398), and other types of heart disease (ICD-9-CM codes 420–429), were acquired by tracking all ambulatory medical care and inpatient records in the NHI database in the year before the index visit. Since hypertension is an important risk factor of HS, we also included the information on the use of antihypertensive medication (e.g. angiotensin converting enzyme inhibitors, alpha blockers, angiotensin II receptor blockers, beta blockers, calcium channel blockers, diuretics, vasodilators, and others) in the analysis. Moreover, we included the information on the use of anticoagulant medication (warfarin) in the analysis because patients undergoing anticoagulant therapy may be at higher risk of bleeding.

### Statistical analysis

The Chi-square test and Student's *t* test were used to examine differences in demographic variables and comorbid medical disorders between the migraine and non-migraine groups. The HS-free survival curves for these two groups were generated using the Kaplan-Meier method and whether the difference in survival between the two groups is statistically significant was assessed using the log-rank test. The Cox proportional hazards regression was used to estimate the effects of the migraine on the risk of HS, with adjustment for demographic characteristics and medical comorbidities. Univariate analysis was initially performed for each variable, followed by stepwise multiple regression analysis. A variable had to be significant at a p value of 0.25 to be entered in the stepwise regression model, while a variable in the model has to be significant at the 0.15 level for it to remain in the model [11]. An alpha level of 0.05 was considered statistically significant for all analyses, which were performed using SAS 9.2 software (SAS Institute, Cary, NC).

## Results

Table 1 shows the demographic and clinical characteristics for the migraine and non-migraine groups. The migraine group had a higher prevalence of hypertension (P<0.0001), hyperlipidemia (P<0.0001), coronary heart disease (P<0.0001), chronic rheumatic heart disease (P = 0.0001), and other heart disease (P<0.0001) than the non-migraine group. There was lack of significant difference in the prevalence of diabetes mellitus (P = 0.4024) and the use of anticoagulant medication (P = 0.7185) between the two groups. Among the 3248 migraine patients who had pre-existing hypertension, 2702 (83.2%) had received antihypertensive medication, while 9711 (80.8%) of the 12024 non-migraine patients with hypertension had received antihypertensive medication.

**Table 1:** Demographic and clinical characteristics of the migraine and non-migraine groups.

	Total sample, N = 125550		
	Migraine group	Non-migraine group	
Variable	N = 20925	N = 104625	P value
Women	14583(69.7)	72915(69.7)	1.0000
Age, y	42.8±15.2	42.6±15.2	0.1608
Hypertension	3248(15.5)	12024(11.5)	<0.0001
Diabetes	1255(6.0)	6119 (5.9)	0.4024
Hyperlipidemia	1664(8.0)	5626(5.4)	<0.0001
Coronary heart disease	1481(7.1)	4506(4.3)	<0.0001
Chronic rheumatic heart disease	107(0.5)	351(0.3)	0.0001
Other heart disease	1538(7.4)	4027(3.9)	<0.0001
Use of anticoagulant medication	35(0.2)	187(0.2)	0.7185

Note: Values are expressed as the mean ± SD or n (%).

**Figure 1:**
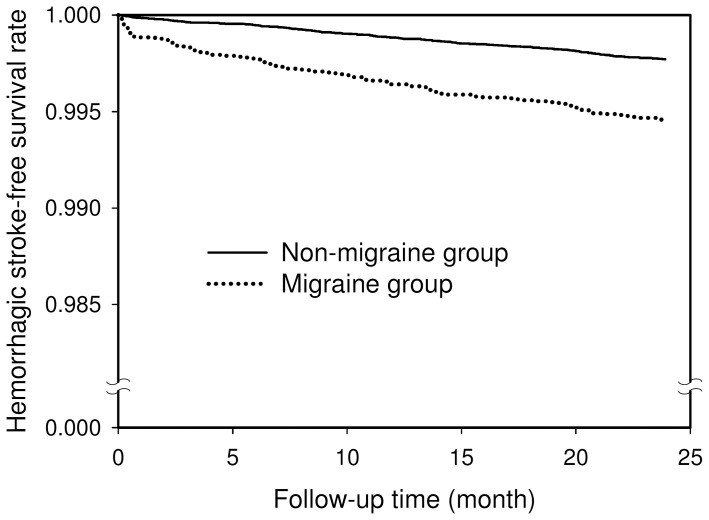
Hemorrhagic stroke-free survival rates for the migraine group (dotted line) and the non-migraine group (solid line).

During the 2-year follow-up, 113 (0.54%) of the 20925 subjects with migraine developed HS compared to 255 (0.24%) of the 104625 subjects in the non-migraine group. Of the 113 HS events in the migraine group, 14 (12.4%) were fatal stroke (death within 30 days after HS onset), while 44 (17.2%) fatal strokes occurred in 255 HS events in the non-migraine group. Comparison of the HS-free survival curves shows that the HS-free survival rate for the migraine group was significantly lower than that for the non-migraine group (log-rank test, P<0.0001, Figure 1).

The results of the Cox regression analysis are shown in Table 2. The left panel shows the crude hazard ratio (HR) for each variable based on univariate analysis. Compared to the non-migraine group, the crude HR of HS for the migraine group was 2.22 (95% CI, 1.78 – 2.77; P<0.0001). Age, sex, hypertension either with or without antihypertensive medication, diabetes, hyperlipidemia, coronary heart disease, chronic rheumatic heart disease, other heart disease, and the use of anticoagulant medication showed significant correlation with the occurrence of HS in the univariate analysis. In the final multiple regression model (the right of Table 2), the adjusted HR of HS for patients with migraine was 2.13 (95% CI, 1.71 – 2.67; P<0.0001) after controlling for other explanatory variables. Other predictors selected in the final model for the risk of HS were age, sex, hypertension either with or without antihypertensive medication, diabetes, and chronic rheumatic heart disease.

**Table 2:** Crude and adjusted hazard ratios (HR) for the occurrence of hemorrhagic stroke during the two-year follow-up period in the migraine and non-migraine groups.

	Occurrence of hemorrhagic stroke		
Variable	Crude HR (95% CI)	‡Adjusted HR (95% CI)	P value for adjusted HR
Migraine (vs. non-Migraine)	2.22* (1.78 – 2.77)	2.13 (1.71 – 2.67)	<0.0001
Age (year)	1.05* (1.04 – 1.06)	1.04 (1.03 – 1.05)	<0.0001
Sex (female vs. male)	0.54* (0.44 – 0.66)	0.62 (0.51 – 0.77)	<0.0001
Hypertension			
With antihypertensive medication (vs. no hypertension)	4.18* (3.34 – 5.25)	1.74 (1.34 – 2.26)	<0.0001
Without antihypertensive medication (vs. no hypertension)	3.44* (2.20 – 5.37)	1.74 (1.10 – 2.75)	0.0181
Diabetes (yes vs. no)	3.24* (2.46 – 4.27)	1.52 (1.14 – 2.04)	0.0046
Hyperlipidemia (yes vs. no)	2.04* (1.48 – 2.83)	NS	NS
Coronary heart disease (yes vs. no)	2.82* (2.07 – 3.86)	NS	NS
Chronic rheumatic heart disease (yes vs. no)	5.40* (2.56 – 11.40)	2.62 (1.24 – 5.57)	0.0120
Other heart disease (yes vs. no)	2.91* (2.12 – 4.00)	NS	NS
Use of anticoagulant medication (yes vs. no)	6.50^†^ (2.43 – 17.42)	NS	NS

* P<0.0001, ^†^P<0.001 in the univariate analysis.

‡ The adjusted hazard ratios were derived from the final multiple regression model.

Abbreviations: CI, confidence interval; NS, non-significant.

The subgroup analysis of the crude and adjusted HRs of HS is presented in Table 3, the subjects in the migraine group being classified into 3 subgroups according to the presence or absence of aura: (1) migraine with aura (MA, ICD-9-CM codes 346.0); (2) migraine without aura (MO, ICD-9-CM codes 346.1); and (3) uncategorized migraine (MU, ICD-9-CM codes other than 346.0 or 346.1). All 3 subgroups had a significantly increased risk of HS compared to the non-migraine group, regardless of the presence or absence of aura. Table 4 shows the subgroup analysis stratified by sex and age. For all sex and age strata, the migraine group had a consistently higher adjusted HR than the non-migraine group. The interaction between migraine and either sex or age was not statistically significant in the multivariate analysis.

**Table 3:** Crude and adjusted hazard ratios of hemorrhagic stroke during the two-year follow-up for the migraine subgroups.

	Migraine subgroups		
	MA subgroup	MO subgroup	MU subgroup
Occurrence of HS	N = 1834	N = 3683	N = 15408
Yes, n (%)	10 (0.55)	18 (0.49)	85 (0.55)
No, n (%)	1824 (99.45)	3665 (99.51)	15323 (99.45)
Crude hazard ratio (95% CI)	2.24 (1.19 – 4.22)†	2.01 (1.25 – 3.25)†	2.26 (1.77 – 2.90)^‡^
Adjusted* hazard ratio (95% CI)	2.22 (1.18 – 4.18)†	1.74 (1.08 – 2.81)†	2.22 (1.74 – 2.84)^‡^

* Variables included in the multiple regression analyses were age, sex, hypertension (with and without medication), diabetes, hyperlipidemia, coronary heart disease, chronic rheumatic heart disease, other heart disease, and the use of anticoagulant medication.

† P<0.05;^ ‡^P<0.0001.

Abbreviations: HS, hemorrhagic stroke; MA, migraine with aura; MO, migraine without aura; MU, uncategorized migraine.

**Table 4:** Crude and adjusted hazard ratios of hemorrhagic stroke for the migraine and non-migraine groups, stratified by sex and age.

	Patient sex*				Patient age†			
	Women		Men		<45years		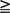 45years	
	Non-MG	MG	Non-MG	MG	Non-MG	MG	Non-MG	MG
Occurrence of HS	N = 72915	N = 14583	N = 31710	N = 6342	N = 61520	N = 12304	N = 43105	N = 8621
Yes, n (%)	145 (0.20)	59 (0.40)	110 (0.35)	54 (0.85)	65 (0.11)	37 (0.30)	190 (0.44)	76 (0.88)
No, n (%)	72770 (99.80)	14524 (99.60)	31600 (99.65)	6288 (99.15)	61455 (99.89)	12267 (99.70)	42915 (99.56)	8545 (99.12)
Crude hazard ratio (95% CI)	1.00	2.04 ‡ (1.50–2.76)	1.00	2.46 ‡ (1.78–3.41)	1.00	2.85 ‡ (1.90–4.27)	1.00	2.00 ‡ (1.54–2.61)
Adjusted hazard ratio (95% CI)	1.00	1.95 ‡ (1.44–2.64)	1.00	2.38 ‡ (1.72–3.30)	1.00	2.58 ‡ (1.72–3.88)	1.00	1.91 ‡ (1.46–2.49)

* Variables included in the multiple regression analyses were age, hypertension (with and without medication), diabetes, hyperlipidemia, coronary heart disease, chronic rheumatic heart disease, other heart disease, and the use of anticoagulant medication.

† Variables included in the multiple regression analyses were sex, hypertension (with and without medication), diabetes, hyperlipidemia, coronary heart disease, chronic rheumatic heart disease, other heart disease, and the use of anticoagulant medication.

Abbreviations: HS, hemorrhagic stroke; MG, migraine group.

‡ P<0.0001.

We also compared the distribution of HS subtypes between the migraine and non-migraine groups. Thirty-three (29.2%) of the 113 HS events in the migraine group, and 49 (19.2%) of the 255 HS events in the non-migraine group were subarachnoid hemorrhage (ICD-9-CM code 430). The migraine group had a higher proportion of subarachnoid hemorrhage in HS events (Chi-square test, P = 0.0337). In addition, we conducted a sensitivity analysis on whether migraine is predictive of fatal or non-fatal HS. The results show that migraine was predictive of non-fatal HS (adjusted HR 2.24, 95% CI, 1.76 – 2.85; P<0.0001). However, there was lack of statistically significant association between migraine and fatal HS (adjusted HR 1.49, 95% CI, 0.82 – 2.73; P = 0.1926).

## Discussion

In the present population insurance registry- based, age- and sex- matched follow-up study, we found migraine was associated with an increased risk of HS. Migraineurs had an approximately two-fold higher risk of HS compared to non-migraineurs (adjusted HR 2.13; 95% CI 1.71 – 2.67). It has been controversial whether migraine is linked to an increased risk of HS. Most previous studies were unable to identify a link between HS and migraine [5], [9], [10], only relatively few studies have reported a positive association between migraine and HS. In an epidemiologic study based on the Dijon Stroke Registry, the frequency of a history of migraine was higher in patients with cerebral hemorrhage (3.6%) and subarachnoid hemorrhage (6.3%) than those with ischemic stroke (1.8%) [7]. Furthermore, a cohort study using data from Women's health study showed that migraine with aura was a risk factor of HS (adjusted HR 2.25, 95% CI, 1.11 – 4.54) [8]. Nevertheless, the mechanism underlying the positive association between migraine and HS is still unclear. We propose the following possible explanations.

Migraine has been linked to dysfunction of cerebrovascular autoregulation [12], which, in turn, has been suggested to be related to occurrence of HS [13]–[15]. Thus, the association between migraine and HS found in our study may be explained, at least in part, by the association between migraine and dysfunction of cerebrovascular autoregulation. In addition, reversible cerebral vasoconstriction syndrome (RCVS), characterized by reversible constriction of the cerebral arteries, has been associated with migraine [16], [17]. Because a higher risk of HS has been reported in patients with RCVS [17], [18], the link between RCVS and migraine may also contribute to the higher risk of HS in migraineurs. In our study, the comparison of HS subtype showed that subjects in the migraine group are more likely to have subarachnoid hemorrhage than the non-migraine group. Because subarachnoid hemorrhage has been considered as a major type of hemorrhagic manifestation in patients with RCVS [17], [19], the predisposition of subarachnoid hemorrhage in migraineurs may further support our hypothesis that the increased risk of HS in migraineurs may be partly mediated by the link between RCVS and migraine.

Hypertension is one of the most important risk factors for HS [20]–[22]. Migraine attacks can be accompanied by increased blood pressure [23], which may also increase the risk of developing HS [20]. Nevertheless, after controlling for the presence of hypertension and the use of antihypertensive medication, the adjusted HR (2.13, 95% CI, 1.71 – 2.67) of HS for migraine remained significant and was similiar to the crude HR (2.22, 95% CI, 1.78 – 2.77), suggesting that the association between migraine and HS is likely to be independent of hypertension.

In the present study, we found that migraineurs had a higher risk of HS irrespective of the migraine subgroup (MA, MO, or MU) and that the HRs were similar among the three subgroups (Table 3). However, in the Women's Health Study [8], MA, but not MO, was shown to be significantly related to an increased risk of HS (HR 2.25, 95% CI 1.11– 4.54, P = 0.024). Further studies are required to investigate whether there is a difference in HS risk between migraine subtypes in different ethnic populations.

Migraine is more prevalent in females, with an estimated female: male ratio of 2–3:1 [1], [2]. Data on the association between migraine and HS in men are very limited. We found that migraine was associated with an increased risk of developing HS in both women and men, as shown in Table 4. Moreover, both younger (<45 years) and older (<$>\raster="rg1"<$>45 years) migraineurs were at higher risk of HS (Table 4), which suggests that the association between migraine and HS is due to mechanisms that are independent of age-related vascular changes.

Table 2 shows that chronic rheumatic heart disease was associated with an increased risk of HS. Patients with chronic rheumatic heart disease might receive anticoagulant prophylaxis for prevention of systemic embolism, and therefore may be expected to have a higher risk of bleeding. Nevertheless, we found that chronic rheumatic heart disease remained an independent risk factor of HS after controlling for comorbidities and the use of anticoagulant medication in the multiple regression analysis (adjusted HR 2.62, 95% CI 1.24 – 5.57).

Since the present study is a large population insurance registry- based cohort study and the temporal sequence between migraine and HS is ordered, this enabled us to establish a temporal association between migraine and HS. Such a temporal relationship is essential for establishing a causal connection. However, several limitations should be acknowledged. First, the diagnosis of migraine, HS, and medical comorbidities in our study was determined by the ICD codes from the NHI claim database and there may be concern about the diagnostic accuracy. However, the Bureau of the NHI has formed different audit committees that randomly sample the claim data from every hospital and review charts on a regular basis to verify the diagnostic validity and quality of care. Accordingly, the NHI claim database is an established research database and has been used in various biomedical research fields. [24], [25] In addition, we used case ascertainment algorithms that required at least two ambulatory medical care visits with a principal diagnosis code of migraine to validate the diagnosis, which might be expected to provide adequate diagnostic accuracy. Second, migraine is often under-recognized and therefore the non-migraine group in our study may include some unrecognized migraineurs. Nevertheless, such potential misclassification is expected to weaken the association between migraine and HS (i.e. bias toward the null). Therefore, the positive association between migraine and hemorrhagic stroke (HS) may be underestimated in our study when considering the potential under-diagnosis of migraine in the non-migraine group. Third, because most of the diagnostic codes of migraine in the NHI claim database were encoded using only the 3-digit ICD-9-CM category number (i.e. code 346) without the subcategory number, the information regarding migraine subtypes (MA or MO) was incomplete. Nevertheless, our subgroup analysis showed a consistently increased risk of HS in the MA, MO, and MU subgroups, further support for an association between both MA and MO and a higher risk of HS. Fourth, due to the inherent limitations of the NHI database, information was lacking regarding lifestyle factors, such as smoking, alcohol consumption, and obesity, which may affect the interpretation of our findings. Finally, most inhabitants of Taiwan are of Chinese ethnicity and it is uncertain whether our findings can be generalized to other ethnic groups.

## Conclusions

The present population-based, age- and sex- matched, follow-up study shows that migraineurs have an increased risk of developing HS. Further studies are required to validate our findings and to investigate the underlying pathophysiological mechanism for the positive association between migraine and HS.
